# E2F2 inhibition induces autophagy via the PI3K/Akt/mTOR pathway in gastric cancer

**DOI:** 10.18632/aging.202891

**Published:** 2021-04-21

**Authors:** Hui Li, Shufen Zhao, Liwei Shen, Peige Wang, Shihai Liu, Yingji Ma, Zhiwei Liang, Gongjun Wang, Jing Lv, Wensheng Qiu

**Affiliations:** 1 Department of Oncology, The Affiliated Hospital of Qingdao University, Qingdao, Shandong, China; 2 Department of Oncology, Qingdao Women and Children’s Hospital, Qingdao, Shandong, China; 3 Department of Emergency Surgery, The Affiliated Hospital of Qingdao University, Qingdao, Shandong, China; 4 Central Laboratory, The Affiliated Hospital of Qingdao University, Qingdao, Shandong, China

**Keywords:** gastric cancer, E2F2, autophagy, metastasis, PI3K/Akt/mTOR pathway

## Abstract

Background: E2F2 is a member of the E2F transcription factor family and has important but not fully understood biological functions in cancers. The biological role of E2F2 in gastric cancer (GC) also remains unclear.

Methods: We examined the expression levels of E2F2 in GC using publicly available datasets such as TIMER, Oncomine, GEPIA, UALCAN, etc., and in our patient cohort, using quantitative real-time PCR, western blotting, and immunohistochemistry. We further investigated the effects of E2F2 on phosphatidylinositol 3-kinase (PI3K)/Akt/mammalian target of rapamycin (mTOR) signaling, autophagy, and the migration and invasion of GC cells by the wound healing assay, Transwell assay and transmission electron microscopy.

Results: E2F2 was highly expressed in both GC tissues and cells compared with normal gastric tissues/cells. High E2F2 expression was associated with poor overall survival (OS). In addition, the expression of E2F2 in GC was strongly correlated with a variety of immune markers. E2F2 overexpression promoted the migration and invasiveness of GC cells *in vitro* through inhibition of PI3K/Akt/mTOR-mediated autophagy.

Conclusion: High E2F2 expression was associated with the characteristics of invasive tumors and poor prognosis. E2F2 also had potential modulatory effects on tumor immunity. We discovered a novel function of E2F2 in the regulation of PI3K/Akt/mTOR-mediated autophagy and the downstream processes of cell migration and invasion.

## INTRODUCTION

Gastric cancer (GC) is the fifth most common malignancy and the third leading cause of cancer-related death [1]. In recent years, the diagnosis and treatment technology of gastric cancer has been greatly improved. But due to local recurrence and distant metastasis problems, the five-year survival rate of advanced GC is still very low. [2]. The molecular mechanism of tumor formation and progression is still unclear, which further complicates effective treatment for GC [3]. In addition, the lack of specific markers that target certain tumor types or disease stages is still an important gap in our understanding of GC treatment [4].

E2Fs are a transcription factor protein family that are major regulators of cell death, cell cycle, cell differentiation and proliferation [5]. The biological role of E2F2 in GC remains largely unknown, although some members of the E2F family, such as E2F1, have been extensively studied [6–9]. E2F2 plays converse roles in the occurrence and progression of tumors. On the one hand, E2F2 can inhibit tumorigenesis by inhibiting cell cycle regulators [10]. On the other hand, it can act as an activator to increase the expression of target genes [11]. The role of E2F2 in GC is worthy of further study. This study aimed to fill the gap in the understanding of the role of E2F2 in the occurrence and/or progression of GC and lay the foundation for the development of novel treatment strategies for GC.

More than 90% of cancer deaths are caused by tumor metastasis, and growing evidence suggests that autophagy is closely associated with tumor metastasis [12, 13]. Autophagy is a ubiquitous physiological mechanism in normal and pathological cells. It is the phagocytosis and degradation process of lysosomes on damaged cell structures, senescent organelles and other biological macromolecules. [14, 15]. This process plays an important role in maintaining cell metabolism balance and internal environment stability [16]. In GC, autophagy is associated with cell migration and invasion, which may involve activation of the phosphatidylinositol 3-kinase (PI3K)/Akt/mammalian target of rapamycin (mTOR) signaling pathway [17]. It is unclear whether E2F2 plays a mediating role in these processes.

Here, we investigated E2F2 expression and mutations in GC patients from The Cancer Genome Atlas (TCGA) and various public databases. Using multidimensional analysis, we evaluated the E2F2-related genomic alterations and functional networks in GC and explored their roles in tumor immunity. Finally, we studied GC cell lines to validate the high expression of E2F2 in GC and examined the function of E2F2 in GC cell invasion, migration, and autophagy. Our data show that E2F2 mediated autophagy through the PI3K/Akt/mTOR pathway, which is a novel role for E2F2 in GC cell metastasis.

## MATERIALS AND METHODS

### ONCOMINE database

The ONCOMINE Database (http://www.oncomine.org) is a cancer microarray database and integrated data mining platform that can classify differential expression levels among common cancer types, corresponding normal tissue and clinical and pathological data [18]. In our study, the transcriptional expression data of E2F2 in different cancer tissues and corresponding adjacent normal tissues were obtained from the ONCOMINE database.

### UALCAN database

UALCAN (http://ualcan.path.uab.edu) is a comprehensive and interactive web resource for analyzing clinical data of 31 cancer types in the TCGA database [19]. In this study, UALCAN was used to analyze the mRNA expression of E2F2 in primary gastric cancer tissues and the relationship between this protein and clinicopathological parameters.

### GEPIA database

The (GEPIA) database for interactive analysis of gene expression profiles (http://gepia.cancer-pku.cn/) is an interactive web-based tool containing 9736 tumors and 8587 normal samples from the TCGA and GTEx projects [20]. In this study, GEPIA was used to verify E2F2 expression in GC.

### LinkedOmics database

The LinkedOmics database (http://www.linkedomics.org/login.php) is publicly available portal that includes multi-omics data from all 32 TCGA Cancer types. It also includes mass spectrometry-based proteomics data generated by the Clinical Proteomics Tumor Analysis Consortium (CPTAC) [21]. The Pearson co-expression network was statistically analyzed using the E2F2 correlation coefficient and was displayed in the form of a volcano map, heat map or scatter diagram. The rank criterion was FDR < 0.05 when 1000 simulations were performed.

### cBioPortal database

cBioPortal includes data from 478 gastric cancer pathology reports from the TCGA [22]. It provides visualization, analysis and download of large-scale cancer genomics data. The E2F2 mutation, copy number variation (CNV) and gene co-occurrence in GC were analyzed by the c-BioPortal tool.

### TIMER database

TIMER (https://cistrome.shinyapps.io/timer/) is a comprehensive resource for systematic analysis of immune infiltration in various cancer types and includes 10,897 samples from 32 cancer types [23]. TIMER used the deconvolution method [24] to infer the abundance of tumor-infiltrating immune cells (TIICs) from the gene expression profile. We analyzed the expression of E2F2 in STAD and the correlation between the expression of E2F2 and the content of immune-infiltrating fluid.

### Protein-protein interaction (PPI) network construction and gene enrichment analyses

The STRING database (https://string-db.org) is a database for online searching of protein interaction relationships [25]. The annotated, visualization and comprehensive discovery database (DAVID) (https://david.ncifcrf.gov/) was used to perform an analysis of the motivational gene ontology (GO) and the Kyoto Encyclopedia of Genes and Genomes (KEGG) [26]. GO enrichment analysis can predict gene function according to biological process (BP), molecular function (MF) and cell composition (CC), while KEGG can be used to analyze the pathway of gene enrichment.

### Tissue microarrays (TMAs)

We collected 60 fresh GC tissues and adjacent non-tumor tissues for immunohistochemistry (IHC). Anti-E2F2 antibody (ab235837) was purchased from Abcam (Shanghai, China) [27]. The Medical Ethics Committee of Qingdao University and the Affiliated Hospital of Qingdao University approved the collection of clinical materials for research purposes.

### Cells and culture conditions

The AGS and HGC27 cell lines were purchased from the cell bank of the Chinese Academy of Sciences. The medium consisted of RPMI-1640 medium containing 10% fetal bovine serum (FBS) (Gibco, NY, USA), placed in an incubator at 37°C and 5% CO_2_ [28].

### Transfection

Cells were transfected with plasmids expressing E2F2 (GV141-E2F2), empty vector (GV141-Vector), small interfering RNAs (siRNAs) against E2F2 (siE2F2; Supplementary Table 1) or negative control (siNC) using Lipofectamine 2000 (Invitrogen, Carlsbad, CA, USA). Plasmid and siRNA were purchased from Denechem (Shanghai, China).

**Table 1:** Basic characteristics of 366 GC patients.

**Variables**	**GC patients (*N* = 366)**
Gender (Male/female)	235/131
Age (years, Mean ± SD)	65.57 ± 10.27
Tumor	
T1	17
T2	81
T3	169
T4	98
Regional lymph node	
N0	114
N1	97
N2	78
N3	70
Metastasis	
M0	325
M1	24
Histologic grade	
1	8
2	129
3	220
Pathologic stage	
1	51
2	124
3	151
4	39

### RNA extraction and qPCR

PCR analysis of mRNA expression was performed as described previously [29]. According to the manufacturer's instructions, use Trizol reagent (Servicebio, HP191402) to isolate total RNA from the cells, and use PrimeScript RT Master Mix reagent (TaKaRa, 00691403) to incubate the diluted RNA on a PCR machine at 42°C for 60 minutes. Incubate at °C for 5min to inactivate the reverse transcriptase and reverse transcribed into cDNA. The PCR amplification conditions are as follows: 10 minutes at 95°C; 40 cycles, respectively, at 95°C for 15 seconds, 60°C for 60 seconds, and 95°C with a temperature increase of 0.3°C every 15s. Use SPSS11.5 software to calculate the significant difference in mRNA expression levels between different samples. The relative amount of target gene was performed using the 2-ΔΔCt method, and GAPDH was used as an internal control. The PCR primers used are listed in Supplementary Table 1.

### Western blotting analysis

Western blotting analysis of protein expression was performed as described previously [30]. RIPA cell lytic reagents containing protease and phosphatase inhibitors (Solarbio, Beijing, China) were used to completely lyse the cells for 30 minutes. The protein concentration was determined by BCA protein assay kit (Beyotime, Shanghai, China). The supernatant containing total protein was then mixed with the corresponding volume of 5x SDS buffer, and the mixture was heated at 95°C for 5 minutes. Then, the protein of each sample was concentrated in the prefabricated gel, and the protein was transferred to the polyvinylidene fluoride (PVDF) membrane using a constant current of 300 mA. Seal the membrane with 5% skimmed milk powder in TBST for 2 hours and incubate with appropriate primary antibody (1pur1000) overnight. The next day, the membrane was washed for 10 minutes three times with TBST. At room temperature, the membrane was incubated with the second antibody bound to HRP for 2 hours and washed with TBST for 3 times for 10 minutes each time. Chemiluminescence kits (Life Technologies, Shanghai, China) were used to observe the bound antibodies under the infrared imaging system (ChemiDoc XRS + of Bio-Rad gel imager. Other antibodies used in this study are listed in Supplementary Table 1. Image J is used to analyze densitometric analysis of western blot detected proteins.

### Electron microscopy

The digested and centrifuged cells were collected in a 1.5ml centrifuge tube. The cells were washed with PBS and the supernatant was discarded. Then the cells were fixed in 2.5% glutaraldehyde (Solarbio, Beijing, China) at 4°C. Then the cells were embedded, sliced, and analyzed by transmission electron microscopy.

### Cell migration and invasion assays

The cells were starved in serum-free RPMI-1640 for 24 hours and then digested with 0.25% EDTA-trypsin. 250 μL serum-free RPMI-1640-treated cell suspension was added to the upper chamber of Transwell insert (Corning Costar), and 600 μL complete medium containing 20% serum was added to the lower chamber. After 24 hours in the incubator, the cells were fixed with methanol for 30 minutes, dried and stained with crystal violet for 20 minutes. The unwashed cells in the upper chamber were gently removed with a cotton swab, then observed under an inverted microscope (Nikon, Tokyo, Japan), and the remaining cells were counted. [31]. The images were analyzed by ImageJ software.

### Wound-healing assay

The cells were inoculated on a 6-well plate and a wound was produced on the monolayer surface of the fused cells with a pipette. The floating cells were washed with PBS, then serum-free medium was added to the well and incubated for 24 hours. From the time the wound was produced, the cell image was captured at different time points within 24 hours. The width of the wound at × 100 magnification was evaluated by microscopy (Nikon, Tokyo, Japan), while the length of the wound was measured at random intervals [31]. Data were analyzed by ImageJ software.

### Statistical analysis

Data are presented as means ± standard deviation. Student’s *t* tests and analysis of variancewere used to assess significance. *P* < 0.05 was considered significant.

## RESULTS

### E2F2 upregulation in GC

**Figure 1:**
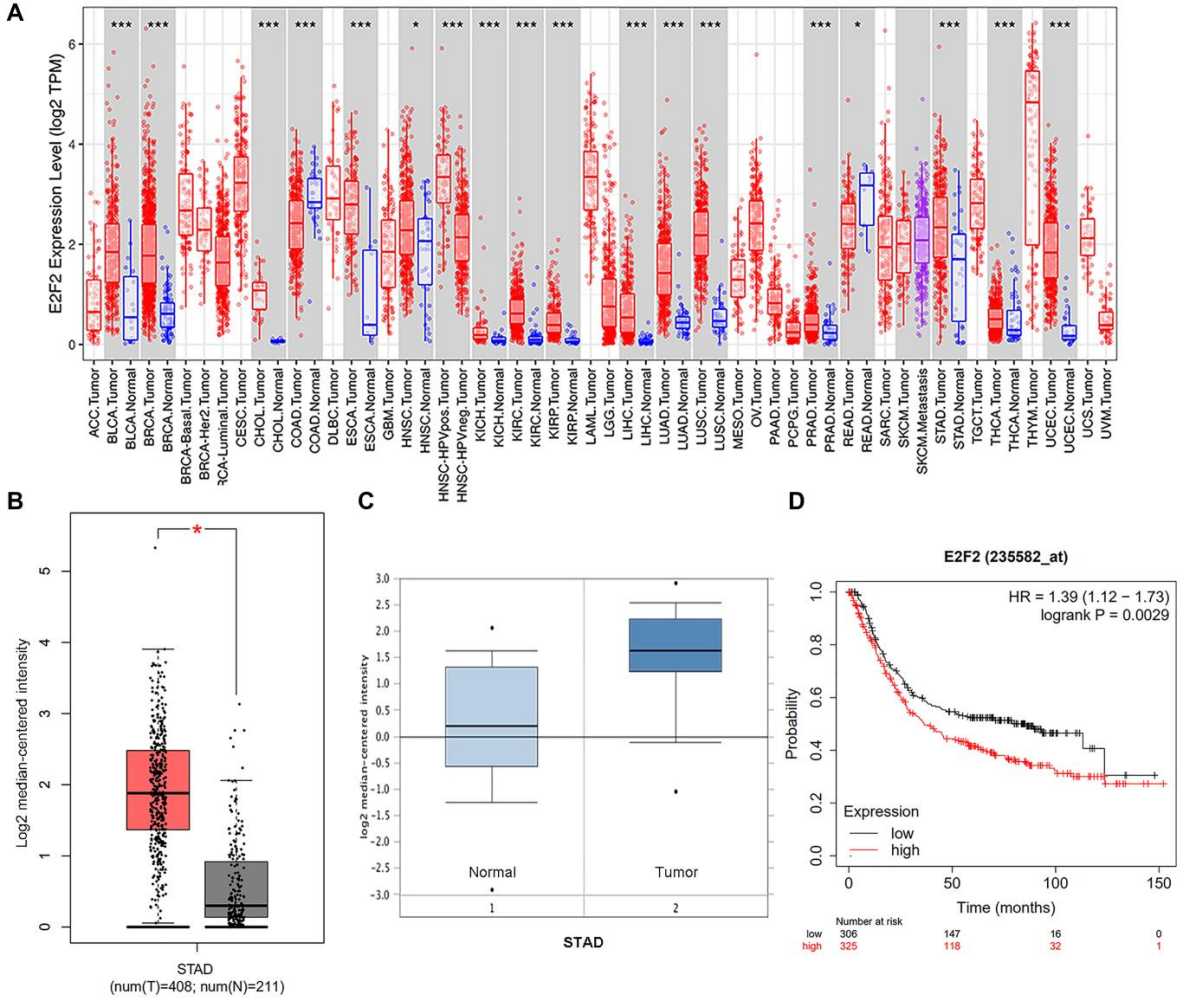
**Expression and methylation of E2F2 in GC tissues and normal tissues as revealed by bioinformatic analysis.** (**A**) E2F2 expression levels in different tumor types from the TCGA database were detected by TIMER (^*^P < 0.05, ^**^P < 0.01, ^***^P < 0.001). (**B** and **C**) E2F2 mRNA is highly expressed in LGG tissues in GEPIA (B) dataset and Oncomine (C) dataset. (**D**) Kaplan-Meier analysis of survival rates of GC patients with high E2F2 expression and GC patients with low E2F2 expression.

**Figure 2:**
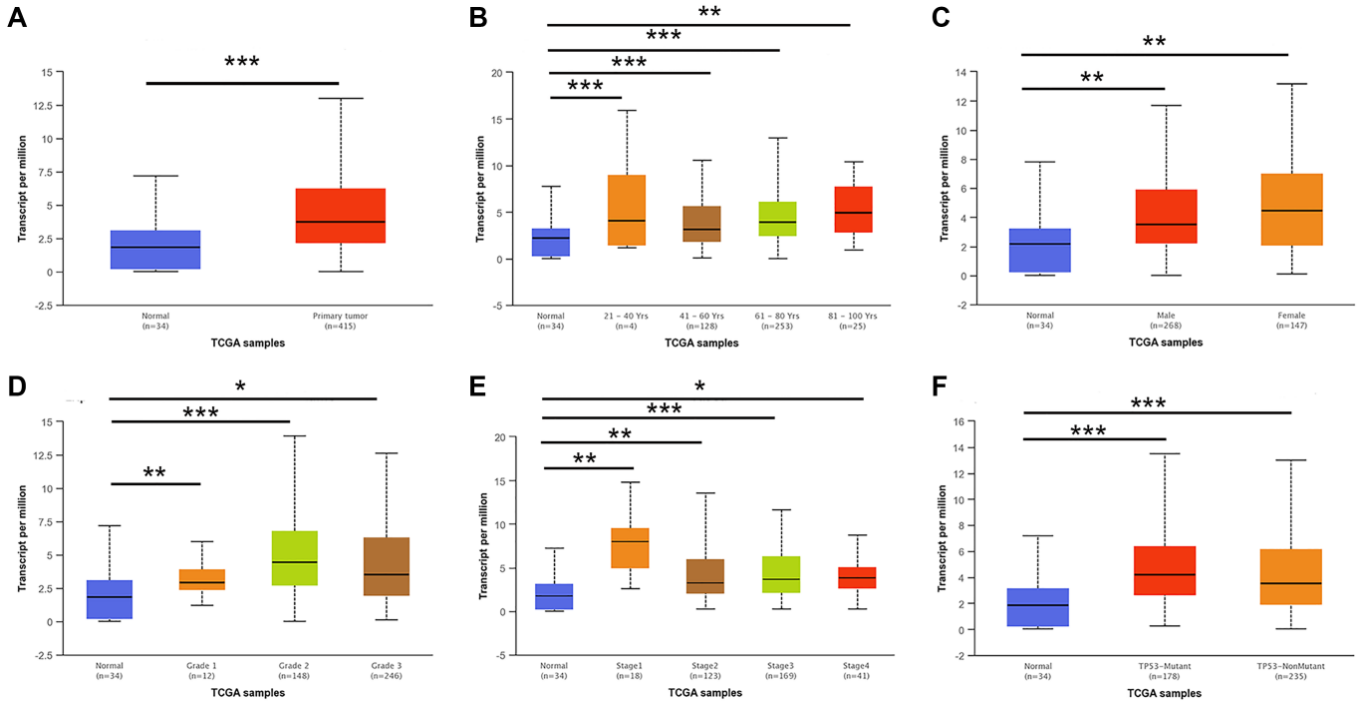
**E2F2 mRNA expression in subgroups of patients with GC, stratified based on gender, age and other criteria (UALCAN).** (**A**) Boxplot shows the relative expression of E2F2 in normal and STAD samples. (**B**) Boxplot shows the relative expression of E2F2 in normal individuals of any age and in STAD patients aged 21-40, 41-60, 61-80, or 81-100 years. (**C**) Boxplot shows the relative expression of E2F2 in normal individuals of either gender and in male or female STAD patients. (**D**) Boxplot shows the relative expression of E2F2 in normal individuals and in STAD patients with stage 1, 2, 3 or 4 disease. (**E**) Boxplot shows the relative expression of E2F2 in normal individuals and in STAD patients with grade 1, 2, 3 or 4 tumors. (**F**) Boxplot shows the relative expression of E2F2 based on TP53 mutation status. The central mark is the median; the edges of the box are the 25th and 75th percentiles. A *t*-test was used to estimate the significance of differences in gene expression levels between groups. ^*^P < 0.05; ^**^P < 0.01; ^***^P < 0.001.

We first detected the expression of E2F2 in various tumor types. After analyzing over a dozen cancers, we found that E2F2 is highly expressed in many tumor types. The expression of E2F2 mRNA in stomach adenocarcinoma was higher than that in paracancerous normal tissues (Figure 1A). We then used Oncomine and GEPIA to analyze E2F2 expression in GC tissues. The results showed that E2F2 was significantly upregulated in GC tissues compared with normal gastric tissues (Figure 1B and 1C). The effect of E2F2 on the overall survival (OS) of GC patients was analyzed using the K-M Plotter. We found that high E2F expression was associated with poor prognosis (Figure 1D). Further subgroup analysis of various clinicopathological features of TCGA-stomach adenocarcinoma (STAD) samples in the UALCAN database consistently showed that the E2F2 mRNA level was elevated. In the subgroup analysis based on sex, age, race, disease stage, and tumor grade, E2F2 expression in GC patients was significantly higher than that in normal controls. In the subgroup analysis of whether TP53 is mutated, the expression level of E2F2 in TP53-mutated gastric cancer patients is higher than that in TP53 non-mutated gastric cancer patients (Figure 2). Therefore, E2F2 might be a diagnostic indicator of GC.

### The independent prognostic value of E2F mRNA expression in GC patients

We downloaded the clinical information of 407 GC patients and the mRNA expression data of E2Fs from the TCGA database (Table 1). The data processed by integration and standardization were analyzed by single-factor and multi-factor Cox regression analysis. Univariate analysis showed that E2F2 expression, age, distant metastasis and pathological stage were closely related to the survival rate (Table 2). In multivariate analysis, high mRNA expression of E2F2 (*P* = 0.0005) and high pathological stage (*P* = 0.036) were closely related to poor OS (Table 3).

**Table 2:** Univariate analysis of overall survival in 366 GC specimens.

**Variables**	**Univariate analysis**		
	**Hazard Ratio**	**95% CI**	***P* value**
Gender	0.775	0.544–1.105	0.160
Age (years)	1.022	1.005–1.039	**0.011**
Tumor			0.109
T(1)	0.098	0.013–0.710	0.022
T(2)	0.768	0.480–1.229	0.271
T(3)	0.882	0.601–1.294	0.521
Regional lymph node			**0.008**
N(1)	0.981	0.134–7.187	0.985
N(2)	1.328	0.182–9.687	0.780
N(3)	1.626	0.222–11.917	0.632
Metastasis			**0.023**
M(1)	0.575	0.282–1.453	0.154
Histologic Grade			0.157
G(1)	0.284	0.030–2.734	0.276
G(2)	0.711	0.221–2.293	0.568
Pathologic Stage			**0.002**
S(1)	0.134	0.032–0.571	0.007
S(2)	0.391	0.191–0.803	0.011
S(3)	0.424	0.202–0.892	0.024
E2F2	0.885	0.792–0.988	**0.030**

**Table 3:** Multivariate analysis of overall survival in 366 GC specimens.

**Variables**	**Multivariate analysis**		
	**Hazard Ratio**	**95% CI**	***P* value**
Age (years)	1.033	1.014–1.052	**0.001**
Regional lymph node			0.300
N(1)	2.002	0.245–16.384	0.518
N(2)	1.459	0.190–11.209	0.716
N(3)	2.004	0.250–16.034	0.512
Metastasis			0.529
M(1)	0.631	0.261–1.526	0.307
Pathologic stage			**0.036**
S(1)	0.114	0.021–0.617	0.012
S(2)	0.29	0.093–0.902	0.033
S(3)	0.439	0.160–1.206	0.010
E2F2	0.841	0.745–0.950	**0.005**

### Co-expression analysis and genomic alterations in E2F2 in GC

**Figure 3:**
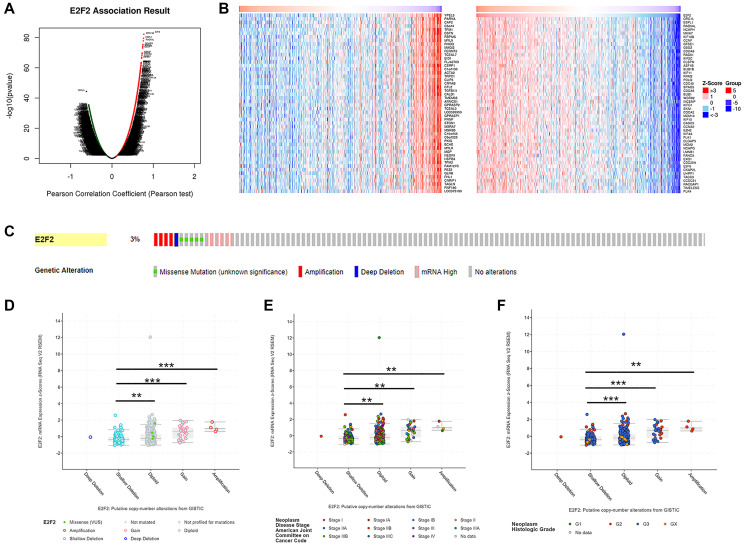
**Genes co-expressed with E2F2 (LinkedOmics) and genomic alterations (cBioPortal) in GC.** (**A**) The global genes highly correlated with E2F2 were identified by Pearson test in the STAD cohort. (**B**) Heat maps show the top 50 genes that were positively and negatively correlated with E2F2 in STAD. Red indicates positively correlated genes and blue indicates negatively correlated genes. (**C**) OncoPrint of E2F2 alterations in the STAD cohort. The different types of genetic alterations are highlighted in different colors. (**D**) E2F2 expression in different E2F2 CNV groups. (**E** and **F**) Distribution of E2F2 CNV frequency in different stage and grade subgroups. ^*^P < 0.05, ^**^P < 0.01, ^***^P < 0.001.

We used the LinkedOmics functional module to assess the co-expression patterns of E2F2 in the STAD cohort. As shown in Figure 3A, 9963 genes (dark red dots) were positively correlated with E2F2 expression and 10262 genes (dark green dots) were negatively correlated with E2F2 expression. The heat map in Figure 3B shows the top 50 genes that have the strongest positive or negative correlation with E2F2. Next, we used the cBioPortal tool to determine the types and frequencies of E2F2 changes in GC based on the DNA sequencing data of STAD patients. E2F2 was altered in 16 (3%) of 473 patients with STAD (Figure 3C). Five cases had missense mutations, 5 cases had high mRNA expression, 4 cases had amplification, and 11 cases had what cBioPortal calls deep deletion. As shown in Figure 3D, compared with the diploid group, the E2F2 expression level in the amplified group was higher. Figure 3E and 3F show the frequency distribution of E2F2 copy number variation (CNV) in groups with different stages and grades, which suggests that the incidence of E2F2 CNV changes was higher in GC and was an early event.

### Relationship among E2F2, immune cells and programmed death-1/programmed death ligand-1 (PD-1/PD-L1)

**Figure 4:**
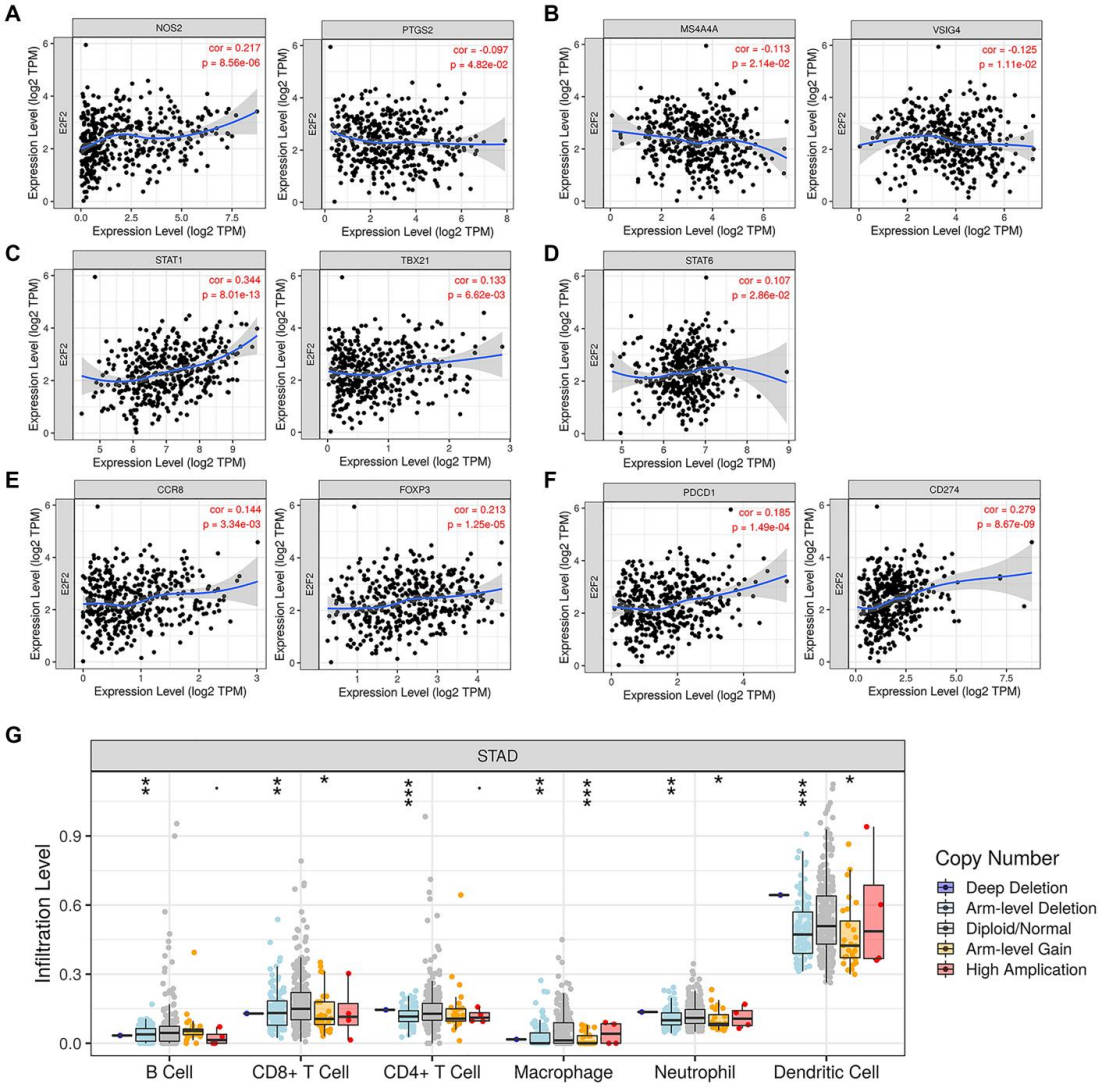
**Expression of E2F2 was related to a panel of gene markers of immune cells,** including M1 cells (**A**), M2 cells (**B**), Th1 cells (**C**), Th2 cells (**D**), Treg cells (**E**) and PD-1/PD-L1 (**F**). (**G**) E2F2 CNV affects the infiltrating levels of B cells, CD8+ T cells, CD4+ T cells, macrophages, neutrophils and dendritic cells in GC.

We searched the TIMER database to assess the correlation between E2F2 mRNA expression and immune cell infiltration. As shown in Figure 4A, after the purity was adjusted, E2F2 expression in GC was significantly associated with M1 cells (Figure 4A), M2 cells (Figure 4B), T helper (Th) 1 cells (Figure 4C), Th2 cells (Figure 4D), and regulatory T (Treg) cells (Figure 4E). In addition, considering the prospect of immunotherapy, we further assessed the relationships between E2F2 expression and PD-1 and PD-L1 (Figure 4F). E2F2 was positively correlated with PD-1 (r = 0.185, *P* = 1.49e-4) and PD-L1 (r = 0.279, *P* = 8.67e-9) expression. Normal tissues served as controls to examine the effect of E2F2 on immune infiltration in different replication states using GISTIC2.0 data. We observed that E2F2 CNV was significantly associated with the degree of infiltration of B cells, CD8^+^ T cells, CD4^+^ T cells, macrophages, neutrophils, and dendritic cells (Figure 4G).

### Construction of the protein–protein interaction (PPI) network and gene enrichment analysis

**Figure 5:**
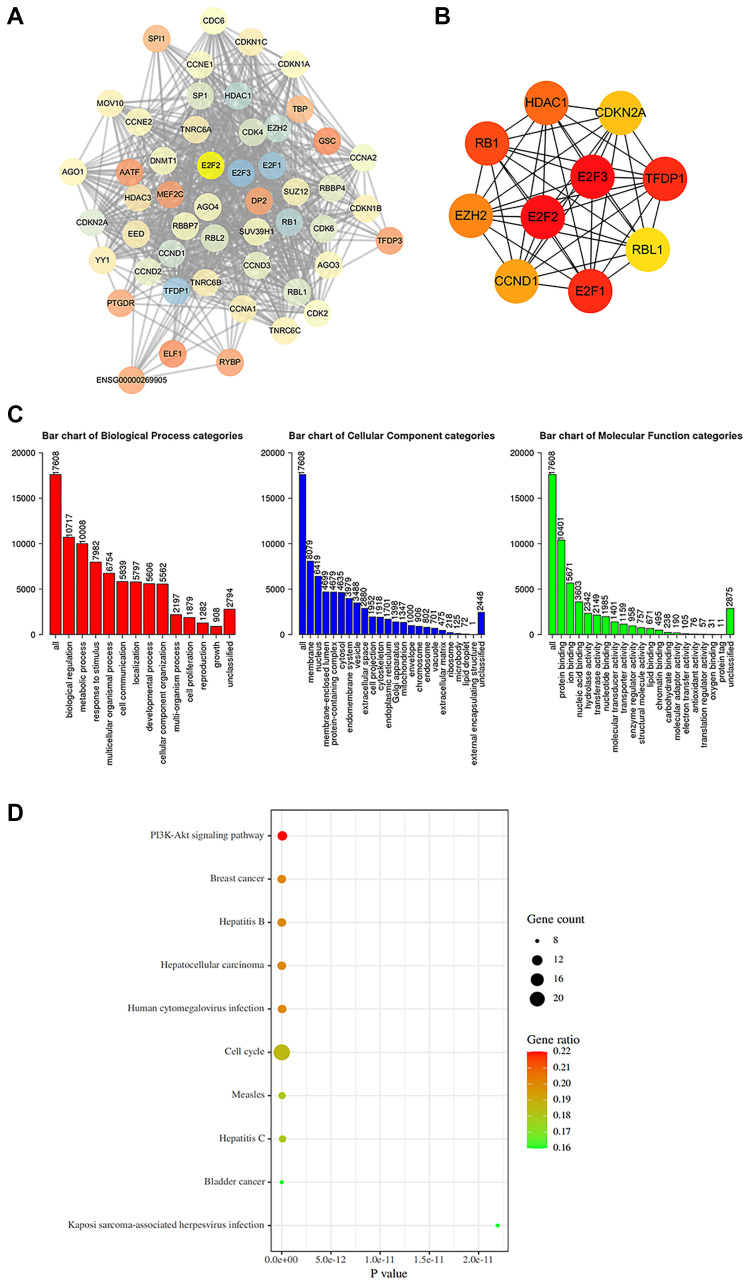
**Protein-protein interaction (PPI) network construction and gene enrichment analyses.** (**A**) Network of E2F2 and its 50 frequently altered neighbor genes was constructed. (**B**) Hub genes were screened from the PPI network using the Closeness, Degree and MCC methods. (**C**) Functional enrichment histogram of important modules. Each biological process, cellular component and molecular function category is represented by a red, blue and green bar, respectively. The height represents the number of IDs in the user list and in the category. (**D**) Pathways enrichment map of E2F2 and its 50 frequently altered neighbor genes. The top 20 terms with the largest number of enriched genes were selected.

To understand the function of E2F2 in GC, we searched the STRING database for proteins that interact with E2F2. We screened 50 genes with the highest correlations and used Cytoscape to construct a complete network (Figure 5A). We found that genes associated with the cell cycle, including cyclin E1 (CCNE1), CCNE2, cyclin-dependent kinase 2 (CDK2), CDK4, CDKN1, and CDKN2A, were closely correlated with changes in E2F2. Figure 5B shows 10 hub genes that are closely correlated with E2F2. Next, the functions of E2F2 and its 50 correlated genes were analyzed using GO and KEGG analyses. GO enrichment analysis showed gene functions in three domains: biological process (BP), cellular component (CC), and molecular function (MF). As shown in Figure 5C, under BP, the E2F2-correlated genes were mainly enriched in biological regulation, metabolic processes, and response to stimuli. Under CC, the genes were mainly enriched in the membranes, nucleus, and membrane-coated lumina. The MFs were mainly protein binding, ion binding, and nucleic acid binding. KEGG analysis (Figure 5D) showed that these genes were most abundant in cell cycle-related pathways, which was consistent with the results of the PPI analysis. The PI3K/Akt signaling pathway also exhibited a high degree of enrichment, which warranted further study.

### E2F2 is a regulator of the PI3K/Akt/mTOR signaling pathway

**Figure 6:**
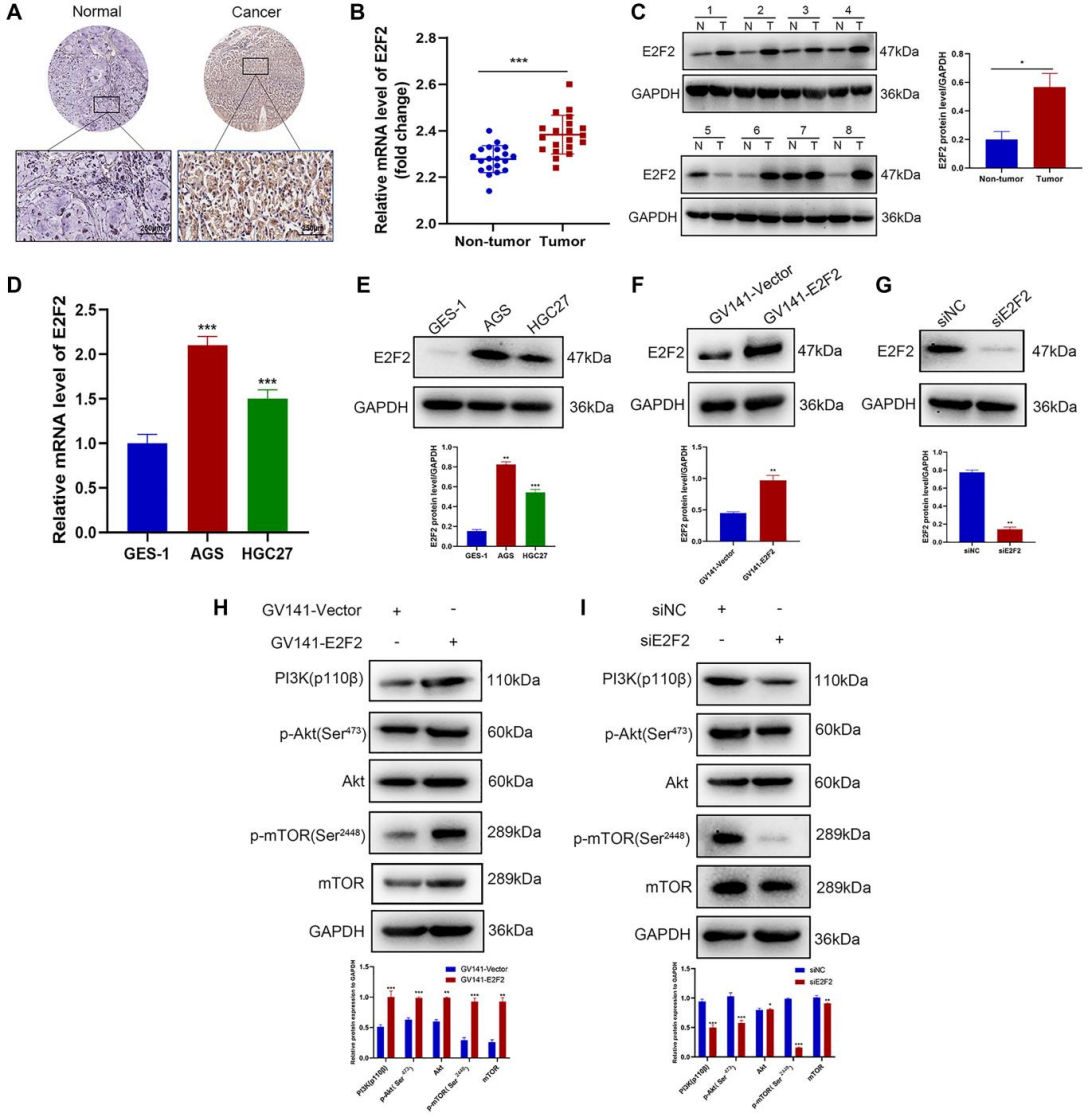
**E2F2 is a regulator of the PI3K/Akt/mTOR pathway.** (**A**) Representative images of E2F2 TMA analysis in GC tissues and adjacent tissues. Scale bar, 250 μm. (**B**) Quantitative PCR (qPCR) analysis of E2F2 mRNA expression in GC and nontumor gastric tissues in our patient cohort. E2F2 mRNA expression levels were normalized to glyceraldehyde 3-phosphate dehydrogenase (GAPDH) expression levels (*n* = 20 per group). (**C**) Western blotting analysis of E2F2 protein expression in GC (T) and nontumor gastric tissues (N). E2F2 protein expression levels were normalized to β-actin expression levels (*n* = 8 per group). (**D**) qPCR analysis of E2F2 basal mRNA expression in three cell lines. E2F2 mRNA expression levels were normalized to GAPDH expression levels. (**E**) Western blotting analysis of E2F2 basal protein expression in the three cell lines; β-actin was used as a loading control. (**F**) Western blotting analysis of E2F2 protein expression in HGC27 cells transfected with GV141-Vector or GV141-E2F2 for 24 h. β-actin was used as a loading control. (**G**) Western blotting analysis of E2F2 protein expression in AGS cells transfected with siE2F2 for 24 h. β-Actin was used as a loading control. (**H**) Western blotting analysis of PI3K 110β, p-AKT, AKT, p-mTOR, and mTOR protein expression in HGC27 cells transfected with GV141-Vector or GV141-E2F2 for 24 h. β-Actin was used as a loading control. (**I**) Western blotting analysis of PI3K 110β, p-AKT, AKT, p-mTOR and mTOR protein expression in AGS cells transfected with siNC or siE2F2 for 24 h. β-Actin was used as a loading control. Data are presented as the mean ± S.D. from three independent experiments. ^*^P < 0.05, ^**^P < 0.01, ^***^P < 0.001.

We sought to further validate the relationship between E2F2 and the PI3K/Akt signaling pathway through *in vitro* experiments. To confirm the high expression of E2F2 in GC tissues, IHC was performed on 60 pairs of GC tissues and paracancerous tissues. Representative images are shown in Figure 6A. The E2F2 expression level in paracancerous normal tissues was significantly lower than that in GC tissues. We performed qPCR (Figure 6B) and western blotting (Figure 6C) on matched GC and adjacent nontumor frozen tissues and observed that E2F2 was significantly overexpressed in GC tissues. The E2F2 expression in cultured GC cells was detected by qPCR and western blotting. We found that E2F2 mRNA (Figure 6D) and protein (Figure 6E) were significantly upregulated in AGS and HGC27 cells compared with GES-1 cells (Figure 6D and 6E). To better understand the impact of E2F2 on the biological behavior of GC, we used GV141-E2F2 and siE2F2 to transfect HGC27 cells and AGS cells, respectively. High overexpression efficiency and gene knockout efficiency were observed in the treated cells (Figure 6F and 6G). Subsequently, we investigated the effect of E2F2 overexpression and knockdown of the PI3K/Akt/mTOR pathway in these cell lines. Western blotting showed that 24 h after forced E2F2 overexpression, PI3K110β, phosphorylated Akt (p-Akt), and phosphorylated mTOR (p-mTOR) expression was increased in HGC27 cells (Figure 6H). In contrast, after E2F2 was knocked down by siE2F2 for 24 h, the expression levels of PI3K110β, p-Akt, and p-mTOR were significantly decreased in AGS cells (Figure 6I).

### Effects of autophagy-mediated E2F2 expression on the migration and invasiveness of GC cells

**Figure 7:**
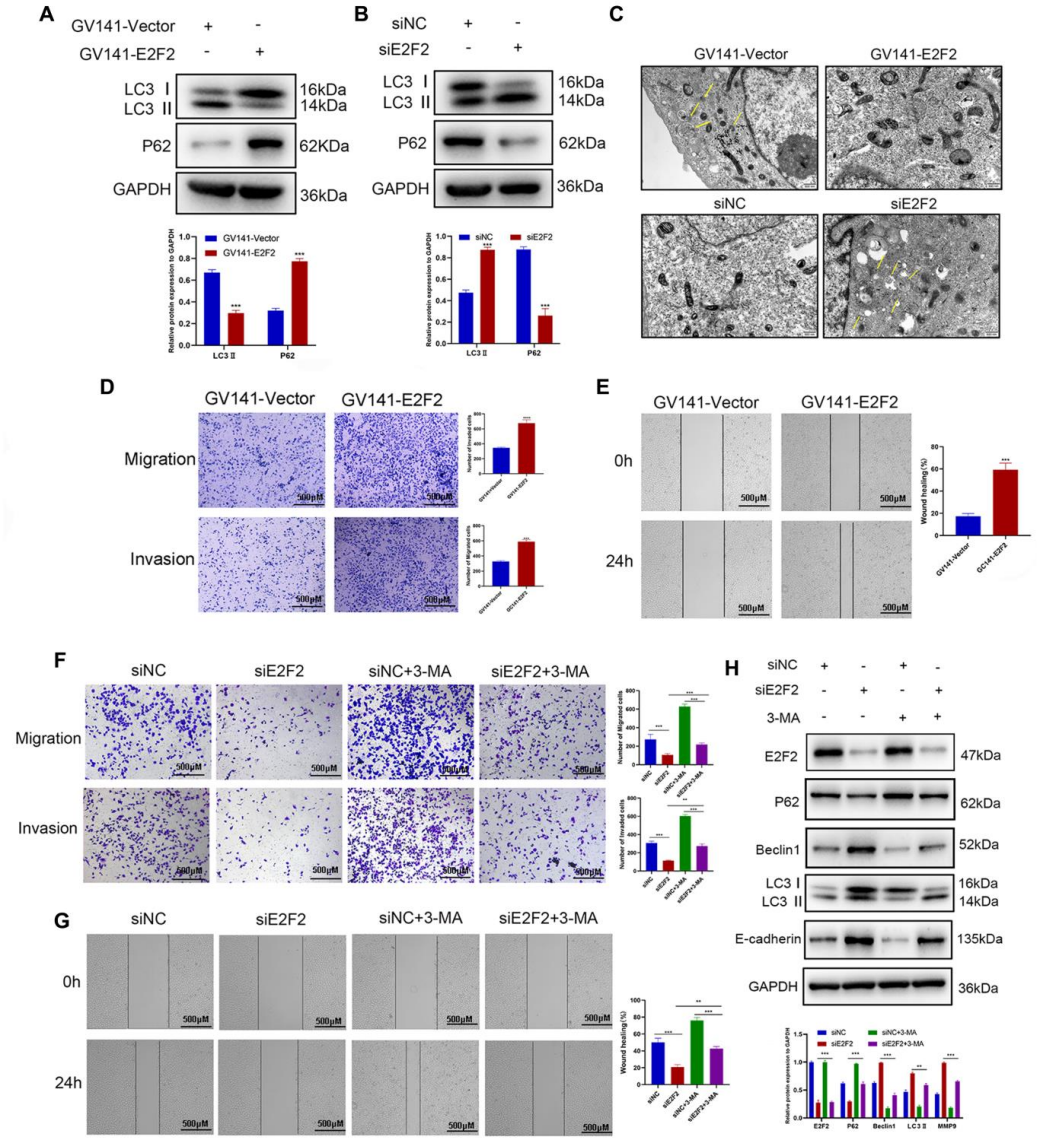
**Effects of E2F2 expression levels on GC cell migration and invasion via autophagy mediation.** (**A**) Western blotting analysis of P62 and LC3-II protein expression in GC cells transfected with GV141-Vector or GV141-E2F2. β-actin was used as a loading control. (**B**) Western blotting analysis of P62 and LC3-II protein expression in GC cells transfected with siNC or siE2F2. β-actin was used as a loading control. (**C**) Representative electron micrographs of autophagic vesicles in GC cells transfected with GV141-Vector or GV141-E2F2 and in GC cells transfected with siNC or siE2F2. (**D**) GC cells were transfected with GV141-Vector or GV141-E2F2 for 24 h. Comparison of GC cell migration and invasion using Transwell compartments. (**E**) A wound-healing assay was performed to compare the motility of GC cells. The wound-healing area was analyzed using ImageJ software. (**F**) GC cells were transfected with siNC and siE2F2 or treated with phosphate-buffered saline (control), 3-methyladenine (2 mM) or a combination of both treatments for 24 h. Comparison of GC cell migration and invasion using Transwell compartments. (**G**) Wound-healing assay was performed to compare the motility of GC cells. (**H**) Western blotting analysis of P62, Beclin1, LC3-II and MMP9 protein expression. β-actin was used as a loading control. Data are presented as the mean ± S.D. from three independent experiments. ^*^P < 0.05, ^**^P < 0.01, ^***^P < 0.001.

Since E2F2 can regulate the PI3K/Akt/mTOR pathway and the PI3K/Akt/mTOR pathway plays an important role in autophagy [32, 33], we further investigated the relationship between E2F2 and the expression of autophagy-related proteins by transfecting GC cells with GV141-EE2F2 or siE2F2. We found that after E2F2 overexpression, LC3II expression was significantly decreased, while p62 protein was significantly increased (Figure 7A). In contrast, after E2F2 inhibition, LC3II was significantly increased, while p62 protein was significantly decreased (Figure 7B). Interestingly, in HGC27 cells transfected with GV141-E2F2, the number of autophagic vesicles was also significantly decreased, while the number of autophagic vesicles was the highest in AGS cells transfected with siE2F2 (Figure 7C). In summary, our data showed that E2F2 overexpression inhibited autophagy in GC cells, and more specifically, inhibited E2F2-induced autophagy. We observed that E2F2 overexpression significantly increased the migration and invasiveness of GC cells (Figure 7D and 7E). When cells were cotreated with siE2F2 and the autophagy inhibitor 3-MA, inhibition of the E2F2-mediated antimetastatic effect was reversed (Figure 7F and 7G). This combined treatment significantly reduced the expression of E2F2 inhibition-dependent LC3-II and Beclin1 proteins and increased the protein expression of p62 and matrix metalloproteinase 9 (MMP9) (Figure 7H). Therefore, our results suggest that E2F2 overexpression promotes GC cell migration and invasion by inhibiting autophagy. Inhibition of E2F2 induces autophagy, thereby inhibiting the migration and invasiveness of GC cells.

## DISCUSSION

The E2F family is a transcriptional regulator in the cell cycle, which plays an important role in cell proliferation, apoptosis, differentiation, DNA damage repair and angiogenesis. E2F2 is an important member of the E2F family and plays a key role in regulating cell cycle and DNA replication [34]. Previous studies have shown that E2F2 plays different roles in different types of tumors. It has been reported that E2F2 plays a carcinogenic role in liver cancer [10] and breast cancer [11], while in prostate cancer, E2F2 inhibits tumor cell proliferation by targeting micorRNA [35]. The role of E2F2 in GC has not been fully confirmed. To understand the potential function of E2F2 in GC and its regulatory network in greater detail, we conducted a bioinformatic analysis on published data and followed this with experimental validation to guide future studies on GC.

Using a large number of independent datasets, we confirmed that E2F2 expression was significantly increased in GC tissues compared with non-tumor gastric tissues. The prognosis of GC patients with high E2F2 expression was worse than that of patients with low E2F2 expression. Consistent with this, our Cox regression model confirmed that the E2F2 expression level was an independent predictive factor for the OS of GC patients. Subgroup analysis was performed on multiple clinicopathological features of TCGA-STAD specimens. Subgroup analysis based on age, sex, tumor grade and disease stage showed that E2F2 expression in GC patients was significantly higher than that in the normal control group. Therefore, our results indicate that E2F2 is upregulated in many GC cases and it is worthy of further study.

A recent study found that genomic alterations often occur in human tumors [36]. CNV may have significant genomic implications, such as interference with gene expression, alteration of genetic content, and induction of phenotypic differences [37]. This study revealed that E2F2 gene copy number was increased in GC tissues and that the major types of E2F2 gene changes were missense mutations and amplifications, which were associated with shorter survival time.

The tumor microenvironment, that is, the internal environment in which tumor cells are produced and lived, is closely related to genome analysis [38]. The tumor microenvironment includes not only the tumor cells themselves, but also the surrounding fibroblasts, inflammatory cells, glial cells and immune cells [39]. In this study, we observed that E2F2 expression in GC tissues was positively correlated with infiltration by M1/M2 cells, Th1/Th2 cells, Tregs, and macrophages. E2F2 regulates the sensitivity of cells to external signals by negatively regulating the AhR pathway of T lymphocytes [40]. Our results show that there is a specific correlation between E2F2 and immune infiltrating cells in GC. The regulation of autophagy in the immune system is a complex and multi-level process. Autophagy has been shown to be an integral part of innate immune responses against microorganisms. The inhibition or activation of autophagy can make the dynamic changes of immune cells to regulate the occurrence and development of tumor. [41]. At present, the relationship between autophagy and T cells, Treg cells, and macrophages has become the main research content [42]. Our research has shown that the expression of E2F2 is related to the above-mentioned immune cells. Whether E2F2 is a key factor in the mediation of immunotherapy and how E2F2 mediates autophagy to regulate tumor immune cells requires further research.

The observed correlation between E2F2 expression and the mTOR pathway enabled us to study the novel role of E2F2 in autophagy. We found that this role was regulated by the PI3K/Akt/mTOR pathway. The overexpression of E2F2 down-regulated the expression of LC3II protein and up-regulated the expression of p62 protein, which inhibited autophagy and promoted the migration and invasion of GC cells *in vitro*. Inhibition of E2F2 increased the expression of LC3II protein and decreased the expression of p62 protein, resulting in the promotion of autophagy and inhibition of migration and invasion of GC cells. LC3 is the first autophagy marker discovered. Its precursor cuts the carboxyl terminus to produce LC3I. Then LC3I covalently binds to the phospholipids on the autophagosome membrane through ubiquitin-like modification to form LC3II. The amount of LC3II is positively correlated with the number of autophagosomes, which is a key indicator reflecting the autophagy activity of cells [43]. As a ubiquitinated substrate, the autophagy receptor protein p62 can be packaged into autophagosomes for degradation. The expression level of p62 protein is inversely proportional to autophagy activity, and is an auxiliary indicator for detecting autophagy activity [44]. In addition, we observed the autophagy inhibitor 3-MA partially reversed the inhibitory effect of E2F2 on GC cell invasion and migration. Previous studies have reported the role of E2F1 in autophagy regulation [45]. E2F2, as a member of the E2F transcription factor family, has no confirmed role in autophagy. Our study is the first to report that E2F2 may play a significant role in autophagy. This finding provides unique ideas into the dual role of E2F2 in cancer cells. E2F2 may regulate the growth and progression of GC in complex ways, which may be achieved through the regulation of autophagy.

## CONCLUSIONS

This study revealed that E2F2 is overexpressed in GC, and the high level of E2F2 expression is related to the malignant biological behavior of the tumor and the poor prognosis of the patients. There is a correlation between the expression of E2F2 and the immune infiltrating cells in GC, which suggests that E2F2 may play a potential role in tumor cellular immunity. Functionally, we found that E2F2 regulates autophagy at least partly through the PI3K/Akt/mTOR pathway, which in turn affects the invasion and migration of GC cells. These findings should be confirmed by large-scale clinical studies and more in-depth mechanistic studies. The current results are encouraging, as our findings may provide novel ideas for the diagnosis and treatment of GC.

### Ethics approval and consent to participate

The collection of samples used in this study was approved by the Ethics Committee of The Affiliated Hospital of Qingdao University.

### Availability of data and materials

All data generated or analyzed during this study are included in the published article. Further details are available from the corresponding author upon reasonable request.

## Supplementary Materials

Supplementary Table 1
