# Supplementary material for: E2F2 inhibition induces autophagy via the PI3K/Akt/mTOR pathway in gastric cancer
Source: Aging (Albany NY). 2021 Apr 21;13(10):13626–43. doi: 10.18632/aging.202891 (PMC8202834; doi:10.18632/aging.202891)
Supplement: Supplementary Table 1 [file aging-13-202891-s001.pdf]

SUPPLEMENTARY TABLE

Supplementary Table 1. Antibodies and primers.

**Antibodies**				
**Name**	**Manufacturer**	**Number**	**Type**	**Usage**
E2F2	abcam	ab235837	Monoclonal	WB, IHC
PI3K p110β	Cell Signaling Technology	#9655	Monoclonal	WB
p-AKT	Cell Signaling Technology	#4060	Monoclonal	WB
AKT	Cell Signaling Technology	#4691	Monoclonal	WB
p-mTOR	Cell Signaling Technology	#5536	Monoclonal	WB
mTOR	Cell Signaling Technology	#2983	Monoclonal	WB
Beclin1	Cell Signaling Technology	#3495	Monoclonal	WB
P62	Cell Signaling Technology	#88588	Monoclonal	WB
LC3	Cell Signaling Technology	#12741	Monoclonal	WB
MMP9	Abcam	ab38898	Polyclonal	WB
GAPDH	Abcam	ab9485	Polyclonal	WB, IHC
goat anti-mouse IgG-HRP	Absin	abs20001	Polyclonal	WB
goat anti-rabbit IgG-HRP	Absin	abs20002	Polyclonal	WB
**siRNAs**	**sequences (5′–3′)**			
E2F2	AACCAGUCAAGGCAAAGUUTT/ UACUUUGCCUUGACUGGUATT			
**Primer sequences (5′-3′)**				
E2F2	F: CGTCCCTGAGTTCCCAACC	R: GCGAAGTGTCATACCGAGTCTT		PCR
GAPDH	F: TGACTTCAACAGCGACACCCA	R: CACCCTGTTGCTGTAGCCAAA		

Abbreviations: WB: Western blotting; IHC: Immunohistochemistry; siRNA: Small interfering RNA
